# Nutrigenomics in livestock—recent advances

**DOI:** 10.1007/s13353-019-00522-x

**Published:** 2019-10-31

**Authors:** Joanna Nowacka-Woszuk

**Affiliations:** Department of Genetics and Animal Breeding, Poznan University of Life Sciences, Wolynska 33, 60-637 Poznan, Poland

**Keywords:** Diet, Farm animals, Gene expression, Epigenetics, DNA methylation, Histone modifications

## Abstract

The study of the effects of nutrients on genome functioning, in terms of gene transcription, protein levels, and epigenetic mechanisms, is referred to as nutrigenomics. Nutrigenomic studies in farm animals, as distinct from rodents, are limited by the high cost of keeping livestock, their long generational distance, and ethical aspects. Yet farm animals, and particularly pigs, can serve as valuable animal models for human gastrological diseases, since they possess similar size, physiology, and nutritional habits and can develop similar pathological states. In livestock, the effects of dietary modifications have mostly been studied with reference to effective breeding and their influence on production traits and animal health. The majority of such studies have looked at the impact of various sources and quantities of fat and protein, supplementation with microelements, and plant-derived additives. The period of life of the animal—whether prenatal, neonatal, or mature—is typically considered when a modified diet is used. This review presents a summary of recent nutrigenomic studies in livestock.

## Introduction

**Fig. 1:**
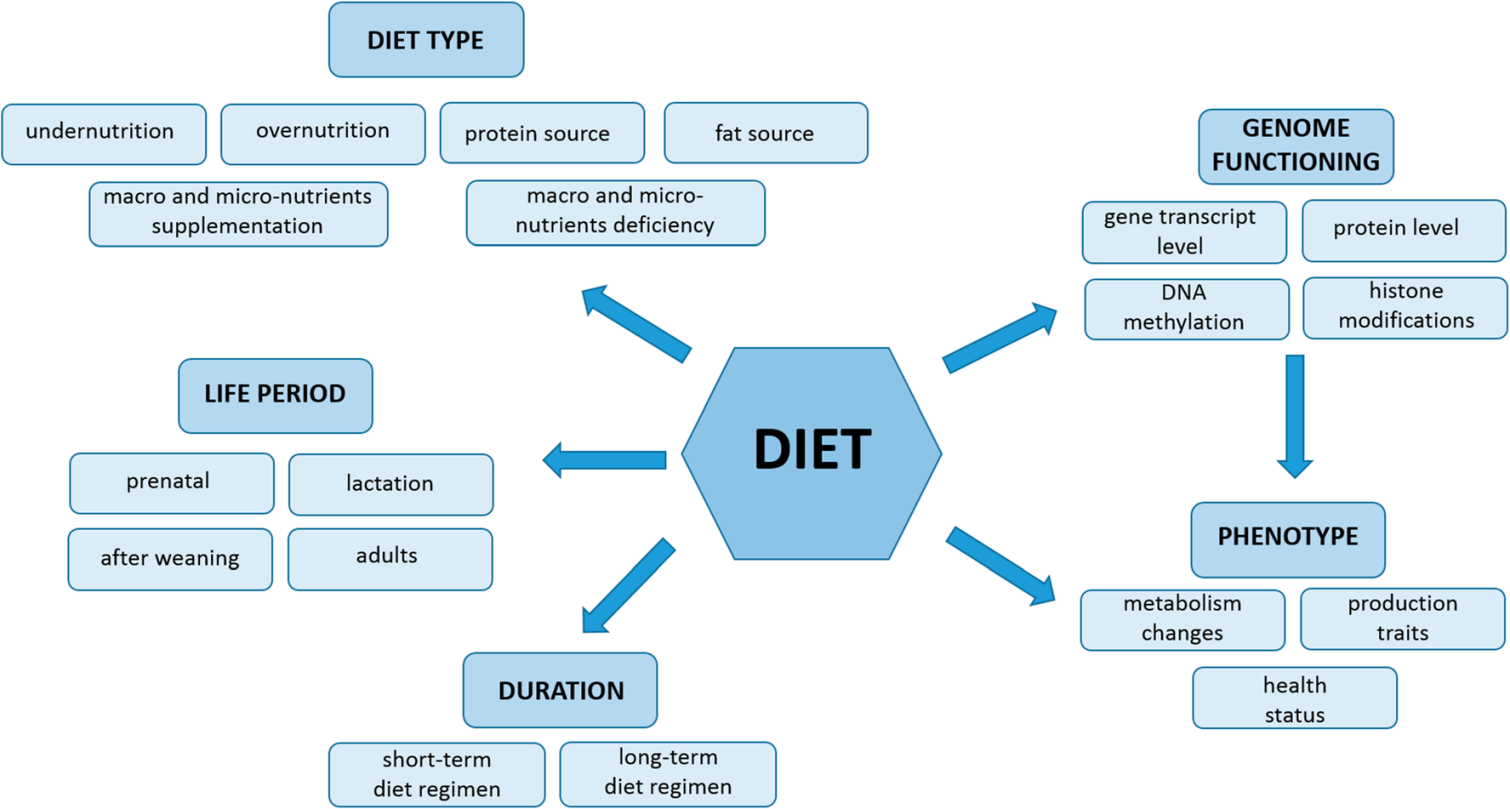
Overview of dietary aspects examined in nutrigenomic studies

Nutrigenomics is presently one of the most rapidly developing research fields; it includes studies on the impact of dietary components on genome functioning in terms of gene expression patterns and epigenetic modifications, such as DNA methylation and histone modifications (Bordoni and Gabbianelli 2019) (Fig. 1). The epigenetic mechanisms, which are very sensitive for nutritional changes, are involved in many basic biological processes including genomic imprinting. The knowledge concerning e.g. the number of known imprinted genes in livestock is very scarce when compared to human or mouse, but the researcher’s interest of such particular genes is still growing, since these genes can play a crucial role in production traits, e.g., maternally imprinted insulin-like growth factor 2 (*IGF2*) gene in pigs contribute to muscle mass and fat deposition (Triantaphyllopoulos et al. 2016). Also the identification of novel noncoding RNAs, e.g., microRNAs in farm animals, is developing. For the last 10 years the number of microRNA precursors reported in the miRBase for cattle and pigs increased three and five times, respectively (Ibeagha-Awemu and Zhao 2015; http://www.mirbase.org/).

The majority of nutrigenomic studies have concentrated on human nutrition and the effects of nutrients on disease etiology (Reddy et al. 2018). Imbalanced diets, whether overnutrition or undernutrition, can have long-term consequences on human health. Overnutrition leading to obesity and cardiovascular diseases is an emerging global problem (Laddu and Hauser 2019). On the other hand, undernutrition leading to deficiencies in calories, macronutrients, and micronutrients can result in serious chronic diseases (de Luca et al. 2017). Experimental studies on the effects of diet on genome functioning have usually been performed on rodent models. Multigenerational experiments and studies of specific life periods (such as pregnancy) have already been performed in mice and rats (Llopis et al. 2014; Kim et al. 2014; Almeida et al. 2015; Chmurzynska et al. 2018). The phenomenon of fetal programming, where maternal nutrition affects progeny genome functioning, has also recently been studied in rodents (Chmurzynska 2010; Ganguly et al. 2014; Nowacka-Woszuk et al. 2017, 2018; Reynolds and Vickers 2018). Similar approaches in farm animals are still in their infancy, but nutrigenomic studies are gaining importance, especially in the meat production industry and for reasons of health protection.

## Nutrigenomics in ruminants

### Prenatal dietary exposure

Beef production worldwide has recently concentrated on improving meat quality in terms of fatty acid profile and marbling. These features have come to be seen as more important due to the effects they can have on human health. Correct lipid profile should improve the levels of conjugated linoleic acid (CLA), of oleic and hypercholesterolemic fatty acids, and the ratio of *n*-6 to *n*-3 polyunsaturated fatty acids (PUFA). Moreover, processes like lipogenesis and intramuscular fat deposition can play a crucial role in marbling, a feature which determines meat quality. Adipogenesis and myogenesis, which begin during early prenatal life, seem to be very sensitive to nutritional factors, and so many studies concerning fetal programming (the effect of the in utero environment, such as the pregnant dam’s diet on the progeny) have also been undertaken in cattle (Ladeira et al. 2018). Muscle and adipose tissues derive from mesenchymal stem cells (MSC), which are controlled by the Wingless and Int (Wnt)/β-catenin signaling pathway. An enhancement of the Wnt signaling pathway promotes myogenesis, while low activity stimulates adipogenesis in skeletal muscle. Undernutrition during the early and middle phases of gestation reduces secondary muscle formation, while nutrient deficiencies in a late gestation decrease the number of intramuscular adipocytes. Both conditions can have a crucial effect on marbling in the progeny (Du et al. 2010; Yan et al. 2013). The peroxisome proliferator-activated receptor-gamma (*PPARG*) gene encodes a key transcription factor involved in adipogenesis. It recently was shown in cattle that maternal overnutrition during pregnancy increases the expression of *PPARG* in the skeletal muscle of fetuses at midgestation, but had no effect on muscle development in the last phase of pregnancy (Gionbelli et al. 2018). Undernutrition in the early and middle phases of gestation resulted in increased adipocyte diameter from subcutaneous and mesenteric adipocyte tissue depots, increased transcription of the fatty acid transporter 1 (*FATP1*) gene in adipose tissue, and reduced the yield grade—an important economical feature of beef carcass quality (Long et al. 2012). Negative energy status (80% of the energy requirements) during midgestation was also studied by Mohrhauser et al. (2015), who noted that such energy deficiencies in progeny could affect fat deposition in intramuscular and subcutaneous fat depots, but without changing muscle mass. The diet of pregnant dairy cattle was also studied for its effect on the progeny. The rumen-protected methionine supplementation around the calving time was tested, and the hepatic expression of genes involved in one-carbon metabolism or transsulfuration pathways was analyzed in the offspring. The liver samples were collected from day 4 to day 50 after birth, and vast changes were seen in the betaine-homocysteine S methyltransferase 2 (*BHMT2*), cystathionine-β-synthase (*CBS*), and adenosylhomocysteinase (*AHCY*) genes. These genes are involved in methionine metabolism and are associated with the level of S-adenosylmethionine (SAM) and S-adenosylhomocysteine (SAH); the ratio of SAM to SAH is an indicator of methylation cell potential. Moreover, the maternal diet reduced the mRNA level of DNA methyltransferase 1 (*DNMT1*) and DNA methyltransferase 3A (*DNMT3A*), consequently leading to DNA methylation alternations (Jacometo et al. 2017). In conclusion, it should be pointed out that dietary manipulation in the specific period of pregnancy can cause different effects, which should be taken into account when comparing individual experimental projects. In addition, in the prenatal period, the nutrient acts not only on the developing fetus, but also on the primary germ cells that will give the next generation, and therefore specific nutrients can have long-term consequences.

### Beef ruminants postnatal feeding: starch source

The effects of a diet on gene expression profile, and thus on meat quality traits, can also be seen in adult animals. Teixeira et al. (2017) studied the effects of different starch administration regimes (whole shelled corn versus ground and silaged corn) on the lipid metabolism gene expression profile and intramuscular fat (IMF) contents of the muscle tissue of Angus and Nellore cattle breeds. They found that, in the Nellore bulls fed ground corn, there was increased expression of the fatty acid binding protein 4 (*FABP4*), acetyl-CoA carboxylase alpha (*ACACA*), and stearoyl-CoA desaturase (*SCD1*) genes. The whole shelled corn in turn increased the expression of the peroxisome proliferator-activated receptor alpha (*PPARA*) gene and reduced the transcription of sterol regulatory element-binding transcription factor 1 (*SREBF1*) gene in both breeds. The authors speculated that the lack of effect of the whole shelled corn diet on marbling was caused by the lower level of *SREBF1*, since this diet reduced the pH in the rumen and increased the contents of linoleic acid (c9, c12–C18:2). The study of Oliveira et al. (2014), where cattle received soybean supplementation, found that the expression of the genes involved in lipid metabolism—such as *PPARA*, *SCD*, *ACACA*, *FABP4*, lipoprotein lipase (*LPL*), and glutathione peroxidase (*GPX1*)—was altered in muscle tissue. On the other hand, alpha-amylase enzyme is often added to beef cattle diets to improve animal performance through improving starch fermentation. A study of this practice was recently described by Elolimy et al. (2018): in this work, finishing steers were supplemented with amylase to determine its effect on performance and on carcass features in relation to liver and muscle global gene expression profiles. In the experimental group, the animals had reduced average daily gain and gain/feed ratio, while no effect was observed on carcass traits or serum metabolites. Muscle tissue showed upregulation of the genes involved in adipogenesis—namely, peroxisome proliferator-activated receptor gamma coactivator 1 alpha (*PPARGC1A*), actin-binding rho activating protein (*ABRA*), and forkhead box O1 (*FOXO1*). The reduced expression of fatty acid binding protein 1 (*FABP1*) and 3-hydroxybutyrate dehydrogenase 1 (*BDH1*) in the liver suggested a potential reduction in hepatic lipid catabolism in the amylase-supplemented animals (Elolimy et al. 2018).

### Beef ruminants postnatal feeding: fat source

Differences in the composition and content of fatty acid in an experimental diet can alter the fatty acid profile in the beef via changes to the lipid metabolism gene expression, which could result in benefits as healthier meat. The effect of oil supplementation on cattle has been studied by Choi et al. (2016), who supplied animals with soybean oil (rich in PUFA) and palm oil (high in oleic acid) on the assumption that the palm oil would promote the expression of adipogenic genes in subcutaneous and intramuscular adipose depots. In animals fed palm oil, the expression of AMP-activated protein kinase alpha (*AMPKα*) decreased in subcutaneous adipose tissue, while the mRNA level of CCAAT enhancer binding protein-beta (*CEBPβ*) reduced in both adipose depots. The soybean oil led to a decreased *SCD* transcript level in the subcutaneous adipose more effectively than did the palm oil, undermining the original hypothesis, which assumed the crucial role of palm oil in promoting adipogenesis (Choi et al. 2016). The *n*-3 PUFA-enriched fish oil diet in cattle also reduced the expression of *SCD* and *SREBF1* genes in muscle, though the expression of both genes was positively correlated with *n*-6 PUFA muscle content (Waters et al. 2009). Differences in the fatty acid content of the diet were tested by Hiller et al. (2011), who compared a grass-silage/*n*-3 fatty acid diet to a maize-silage/*n*-6 fatty-acid-based control diet. They examined the patterns of muscle and adipose tissue expression of lipid metabolism genes in German Holstein bulls. In both tissues, the ratio of *n*-6 to *n*-3 fatty acids decreased and was associated with the downregulation of the *SREBP1*, *ACACA*, fatty acid synthase (*FAS*), and *SCD* genes in muscle and reduced mRNA levels of *ACACA* and *FAS* in adipose tissue. The study of Byrne et al. (2005) determined the entire transcriptome in a muscle tissue of Brahman-cross cattle determined by expression microarray approach. The animals were fed a lucerne hay diet differing in terms of animal growth rate. The variant diets used were high (growth rate from 0.8 to 1.0 kg/day), medium (weight gain of 0.3 kg/day), and low (weight loss of 0.3 kg/day). The restricted diet induced upregulation of 29 genes involved in protein turnover, cytoskeleton structure, and metabolic homeostasis processes, while 28 genes showed reduced transcription levels (these were genes from the extracellular matrix, as well as cytoskeleton structure pathways). Such genes can affect connective tissue remodeling and muscle atrophy, which can in consequence be crucial for meat quality (Byrne et al. 2005). However, it should be noted that the microarray technology used to analyze gene expression provides sometimes inconsistent results. Moreover, the small research groups in this study is a limitation of the presented results. Recently it was shown that supplementation with vitamin E, in the form of alpha tocopherol, in lambs of the Aragonesa breed altered transcript level of *SREBF1* and *PPARG* in muscle and adipose tissue, respectively (González-Calvo et al. 2014). Broad studies of the liver and muscle transcriptome in lambs have also been undertaken in response to dietary supplementation with essential oils extruded from cinnamon bark, dill seed, and eucalyptus leaves (Sabino et al. 2018a). The RNA-seq analysis revealed a sex-dependent effect of the essential oils on the gene transcription in both tissues. The differentially expressed genes were mostly involved in inflammatory and immune response pathways.

### Dairy ruminants postnatal feeding: fat source

The diet of a dairy dam can have a crucial impact on milk yield, as well as on its protein and fat composition. It has been shown that diet-induced milk fat depression (MFD), resulting in decreased milk fat synthesis in the mammary gland, can be caused by specific fatty acids delivered with the diet, especially *trans*-10, *cis*-12 CLA (Bauman et al. 2011). In MFD syndrome, the expression of key lipogenic genes is altered, including *SREBF1*, *FAS*, *LPL*, *ACACA*, and thyroid hormone-inducible hepatic protein (*THRSP*) (Peterson et al. 2003; Harvatine et al. 2018). Similar observations have been made in dairy ewes with MFD, where the mammary expression of lipogenic genes was altered (Carreño et al. 2016; Toral et al. 2017). Recently, there have been studies in cattle and goats of supplementing the diet with different oils—such as fish oil and sunflower oil with starch additions. The mammary expression of lipogenic genes, as well as the milk’s fatty acid composition, was determined. Significant differences were observed in the milk fat content and yield for both species; however, the studied diet types did not alter the expression levels of the analyzed genes, and only species-specific differences in mRNA profiles were found (Bernard et al. 2017; Fougère et al. 2018; Fougère and Bernard 2019). In the study of Faulconnier et al. (2018), linseed oil, alone or with the addition of fish oil, was administered to dairy goats. Both dietary regimens altered the milk’s fatty acid composition; however, the mammary gland expression of selected candidate lipogenic genes was not altered. On the contrary, the global transcriptional profiling showed differences for genes involved in protein metabolism and transport in response to diet supplementation. Dirandeh et al. (2016) also studied dietary oil additives in three variations: (a) diet with whole soybean (rich in *n*-6 PUFA); (b) diet with linseed (rich in *n*-3 PUFA); and (c) diet with palm oil. The hepatic transcript level was determined for growth hormone-receptor 1A (*GHR1A*), insulin receptor (*INSR*), insulin-like growth factor 1 (*IGF1*), and insulin-like growth factor binding protein (*IGFBP2*). It was found that the linseed oil significantly increased the mRNA level of *INSR* and *GHR1A* as compare to other groups, while the same diet elevated the plasma IGF1 concentrations, suggesting an important role of *n*-3 PUFA in regulating somatotropic axis genes. In the study of Mobuchon et al. (2017), the effect of adding sunflower oil to diet was studied in order to see if changes occurred in the expression level of small noncoding RNAs (microRNA) in the mammary gland of lactating cows. MicroRNAs are involved in basic post-transcriptional regulation mechanism, leading to the silence of target genes. Among the 272 identified microRNAs, two (miR-142-5p and miR-20a-5p) were downregulated by sunflower oil supplementation. It was also determined that the potential target genes for these microRNAs included genes associated with lipid metabolism, such as the lipin 1 (*LPIN1*), acetyl-CoA acetyltransferase 1 (*ACAT1*), *PPARA*, *PPARG*, and fatty acid elongase 6 (*ELOVL6*) genes (Mobuchon et al. 2017). This shows that the gene expression analysis should not only focus on the gene transcript level itself, but should also include other mechanisms, such as the level of small RNAs that may be involved in regulating gene expression profile. An analysis of microRNA under dietary regimens has also been conducted in Canadian Holstein dairy cows (Li et al. 2015). At mid-lactation, the animals were supplemented with linseed or safflower oils and the microRNA profile was determined in a mammary gland tissue. The RNA-seq approach allowed 321 known bovine microRNAs to be identified, of which 176 were novel. Linseed oil altered the expression of 14 microRNAs, and safflower oil altered 22. Of these, seven microRNAs (bta-miR-199c, bta-miR-199a-3p, bta-miR-98, bta-miR-378, bta-miR-148b, bta-miR-21-5p, and bta-miR-200a) were altered by both oil types. The analysis of target genes for these microRNAs identified that they may be involved in general cellular metabolism, as well as in lipid metabolism pathways (Li et al. 2015). Moreover, the both oil types significantly reduced the milk fat percentage by approximately 30%. In a later study of the same research group, using the above experimental design with linseed or safflower oil supplementation, the entire mammary gland transcriptome was analyzed (Ibeagha-Awemu et al. 2016). It was found that over 1000 genes had altered gene expression in response to linseed oil, while almost 200 were altered by safflower oil. The greatest changes in both treatments were found for five genes—fructose-1,6-bisphosphatase 2 (*FBP2*), uncoupling protein 2 (*UCP2*), TGFB-inducible early growth response protein 2 (*TIEG2*), angiopoietin-like 4 (*ANGPTL4*), and aldehyde dehydrogenase 1 family member L2 (*ALDH1L2*). These can be considered candidates for milk fat traits. In the same animals, the transcription of genes involved in epigenetic mechanisms (such as those encoding DNA methyltransferases, histone acetylases, deacetylases, and methyltransferases) was studied in the mammary gland of dairy cattle (Li and Ibeagha-Awemu 2017), demonstrating that linseed oil supplementation reduced the expression of the histone deacetylase 2 and 3 (*HDAC2* and *HDAC3*), euchromatic histone lysine methyltransferase 2 (*EHMT2*), lysine acetyltransferase 2A (*KAT2A*), and sirtuin 2 (*SITR2*) genes. This suggests that epigenetic marks, such as histone modification changes, may be involved in the regulation of fatty acid synthesis, thus searching for general mechanisms regulating gene expression, altered by nutrients, should be undertaken.

## Nutrigenomics in pigs

### Protein-restricted diet and protein sources

The pig production industry is one of the largest sources of meat. Nutrigenomic studies of swine have mostly concentrated on the genes involved in the functioning of the muscle, adipose, intestine, and liver tissues (Loor et al. 2015). A study involving a low protein diet with different sources of fat (palm kernel oil, soybean oil, and palm oil) was undertaken by Doran et al. (2006) with a hybrid Duroc × Large White × Landrace pig line. They studied the activity and expression of a key lipogenic gene, *SCD*, in muscle and subcutaneous adipose tissues. They also determined the protein levels of ACACA and FAS, major enzymes involved in de novo fatty acid synthesis. Monounsaturated fatty acid (MUFA) levels and total fatty acids were elevated in muscle tissue in response to protein restriction. The intramuscular fatty acid level was positively correlated with the SCD protein level. The protein-reduced diet also increased the expression of ACACA and FAS proteins, but the oil type only affected ACACA, which increased in animals supplemented with palm oil (Doran et al. 2006). Protein restriction was also used in finishing Duroc gilts to determine the muscle tissue transcriptome. The expression microarray results showed that a protein deficiency in the last feeding period improved very important traits, such as IMF, in muscle tissue, by altering the expression of genes involved in lipid biosynthesis and degradation. The transcript level of adipokines, including leptin (*LEP*), tumor necrosis factor-alpha (*TNFα*), and hypoxia inducible factor 1 subunit alpha (*HIF1α*), also increased. On the other hand, the tested diet could also reduce the capacity for efficient growth by repressing cell cycle and cellular growth, since the genes involved in the protein synthesis pathway were altered. However, this conclusion was based only on the prediction and the fact that the studied genes were assigned to this particular biological pathway (Hamill et al. 2013). Studies of dietary protein source have already been undertaken in pigs. Schwerin et al. (2002) studied the effect of diets based on soy or casein protein isolates, finding that a soy-based diet altered the transcript level of genes involved in oxidative stress response in the liver. Later studies of the same research group, analyzing the hepatic proteome, showed that a soy-rich diet induced changes in protein biosynthesis at the same *loci* where transcriptional changes were already found (Junghans et al. 2004) indicating that both transcript and protein levels should be tested simultaneously to give a chance for solid conclusions. Roh et al. (2015) also investigated soybeans as the major source of protein in pig diet; their hypothesis was that the use of fermented soybeans instead of dry soybeans could modulate the immune response in piglets exposed to immune challenge with a lipopolysaccharide injection. The transcriptional profile was established in leukocytes using oligonucleotide microarray analysis. It was found that 40 genes showed differences in expression, with 17 genes being upregulated and 23 downregulated. These genes were mostly involved in reducing the inflammatory response in growing piglets after the lipopolysaccharide challenge, which suggests potential benefits in growth rate in animals fed with fermented soybeans (Roh et al. 2015).

### Fat sources in pig nutrition

Many studies of fat sources have been undertaken in pigs. One study looked at the supplementation of finishing pigs with CLA in the context of proteome changes in the muscle tissue, as well as IMF content (Zhong et al. 2011). It was shown that CLA in the diet increased IMF and elevated the level of proteins involved in energy metabolism, fatty acid oxidation and synthesis, and amino acid metabolism. Óvilo et al. (2014) studied the effects on fatty acid composition, lipid metabolism, and gene expression patterns in Iberian pigs of different MUFA energy sources, such as high-oleic sunflower oil as an exogenous source of MUFA (HO diet) and carbohydrate diet providing substrates for endogenous MUFA synthesis (CH diets). They noted that the fatty acid profiles in adipose, liver, and muscle tissues reflected the dietary treatment, with the higher MUFA and lower saturated fatty acids (SFA) of the HO diet. Gene expression determined by microarray technology indicated 37 differentially expressed genes in the adipose tissue, while the more accurate qPCR method confirmed significant differences for only three genes: *LEP*, malic enzyme 1 (*ME1*), and retinoid X receptor gamma (*RXRG*). The diet was found to have no effect on the selected gene expression profile for muscle and liver tissues. In a later study by the same research group (Benítez et al. 2017), the HO diet was compared to the CH diet, considering breed effect (Iberian and Duroc pigs) and feeding versus fasting status. The transcript level of selected genes involved in adipogenesis, lipogenesis, and lipolysis in the adipose tissue was analyzed. In both breeds, the HO diet induced MUFA and decreased SFA contents in adipose tissue. The Iberian breed showed higher levels of SFA and lower levels of PUFA than the Duroc breed. The transcript levels of *LEP*, *ME1*, *SCD*, and *ELOVL6* showed significant changes by breed. The feeding status altered the mRNA of the *LEP*, *ME1*, *SCD*, *ACACA*, *ELOVL6*, *RXRG*, *PPARG*, *FASN*, and G0/G1 switch 2 (*G0S2*) genes while feeding type HO versus CH influenced the transcription only for a single perilipin 1 (*PLIN1*) gene in the adipose tissue (Benítez et al. 2017). Recent work by this group (Benítez et al. 2019), in which RNA-seq was used for global transcriptomic analysis of pig subcutaneous adipose tissue in Iberian and Duroc breeds, showed the strong breed effect on the transcript level in terms of growth, extracellular matrix formation, carbohydrate and lipid metabolism, and immune response. The dietary effect was more intense in Iberian breed, changing the transcription of genes associated with inflammation, immune response, lipid metabolism, and fattening. The authors suggested that this breed specific differences may be due to structural variants (e.g., in glucose response elements) located near gene promoter that are sensitive to nutrients and can modulate gene expression. Therefore, the search for long noncoding RNAs responsible for its regulation should be considered (Benítez et al. 2019). Diets with different fat sources (rapeseed oil, beef tallow, or coconut oil) and with the addition of dried distillers’ grains with solubles as a source of protein were studied in adult pigs by Oczkowicz et al. (2016). They determined the whole liver transcriptome by RNA-seq and found that, depending on fat source, altered expression was seen for the genes associated with lipid metabolism—namely apolipoprotein A4 (*APOA4*), acyl-CoA synthetase long chain family member 5 (*ACSL5*), cytochrome P450 2B22 (*CYP2B22*), and glutathione S-transferase omega 1 (*GSTO1*). The highest expression levels of *APOA4*, *CYP2C49*, and *CYP2B* were found in the group receiving coconut oil, while beef tallow had the greatest effect on the transcript level of the *GSTO1* gene (Oczkowicz et al. 2016). In a later study of PUFA supplementation (linoleic acid and α-linolenic acid rich in *n*-6 and *n*-3, respectively) in adult pigs, the liver transcriptome was also studied. It was found that over 3500 genes showed altered expression profiles, with an increased level of expression in the case of genes involved in fatty acid activation, fatty acid β-oxidation, lipid transport activity, and lipid binding; the genes that saw decreased expression were mostly associated with prostaglandin biosynthesis or peroxidase activity. Moreover, the hepatic lipid profile was altered in the experimental group receiving PUFA supplementation. The level of *n*-3 fatty acids was increased while the *n*-6 fatty acids content dropped, leading to a reduction in the *n*-6 to *n*-3 fatty acid ratio (Szostak et al. 2016).

### Supplementation with vitamins and minerals

Correct dietary composition in terms of macroelements and microelements is essential for balanced growth. The effect of dietary supplementation with L-carnitine in piglets was studied by Keller et al. (2011). Carnitine is important for proper energy homeostasis, which regulates the content of acetyl-CoA in cytosol and mitochondria. It improves protein accumulation and muscle mass. The growing piglets were fed a diet supplemented with L-carnitine for 3 weeks and the muscle transcriptome was determined. The authors found that, of the 211 genes differentially expressed, many involved in the insulin-like growth factor (IGF) binding and insulin receptor binding pathways were upregulated, while genes related to proapoptopic and atrophy functions were silenced. This strongly suggested that the L-carnitine can have positive effects on skeletal muscle mass in growing piglets (Keller et al. 2011). Other amino acid supplementation diets have also been tested in pigs, including glutamine supplementation. Glutamine is essential for proper cell division, as it is required for purine and pyrimidine synthesis and is a key substrate for the synthesis of other endogenous amino acids, such as arginine. In a work of Wang et al. (2008), a glutamine-rich diet was tested in weaned piglets to determine whether this nutrient can prevent intestinal dysfunction and atrophy, which is often observed when the feeding type is changing from sucking to chow. The transcriptome of the intestinal tissue was determined, and it was found that the piglets from the experimental group showed increased expression of genes important for cell growth and removal of oxidants. Moreover, the genes responsible for immune activation were downregulated. The glutamine-supplemented diet positively affected small intestine growth, body weight gain, and intestinal oxidative–defense capacity (Wang et al. 2008). Threonine is another amino acid crucial in gut physiology, and a study employing a threonine-deficient diet (30% reduction) was carried out in young piglets for 2 weeks by Hamard et al. (2010). The analysis of the transcriptome of ileal tissue showed that threonine deficiency altered the expression of 324 genes involved in immune and defense responses, protein synthesis, energy metabolism, and other functions. It was also found that these animals had increased paracellular permeability and glucose absorption capacity, indicating that threonine may be crucial in maintaining intestinal integrity (Hamard et al. 2010). However, it must be pointed out that the mentioned above diet regimens were usually tested for a short time and the consequences for longer supplementation were not considered. Contrary to previous reports, long-term supplementation with selenium in adult pigs was carried out by Song et al. (2013) with the aim of determining transcriptome changes in leukocytes. Selenium is a dietary microelement that is necessary for proper thyroid hormone metabolism, as well as the functioning of the immune system and neural cells. The obtained results showed that the transcriptional level of genes associated with both innate and acquired immunity increased, suggesting that long-term selenium supplementation may improve immunity in pigs (Song et al. 2013). A similar study was also recently performed by Sirri et al. (2018) in adult pigs, where three different experimental diets were tested: (a) a diet enriched in *n*-3 PUFA from extruded linseeds; (b) a diet with vitamin E and selenium added as antioxidants; and (c) a diet with an additional source of polyphenols from extruded linseeds and plant extract. RNA-seq established the muscle transcriptome under these three feeding regimens. The results indicated that the PUFA-rich diet downregulated genes involved in muscle development and contraction. Supplementation with vitamin E and selenium reduced the transcription of genes involved in oxidative phosphorylation, suggesting that the reactive oxygen species (ROS) induced by *n*-3 PUFA were eliminated by the antioxidant role of vitamin E and selenium. The diet with additional polyphenols seemed to have a major effect on genes involved in muscle development, as the expression of genes associated with lipid metabolism and immune system was increased (Sirri et al. 2018). These observations were confirmed in a later study by the same research group (Vitali et al. 2019). Recently, the yeast-derived mannan-rich fraction was tested in piglets (Fouhse et al. 2019); the mannan is considered an antioxidant and an antiproliferative polysaccharide that plays a protective role against gastrointestinal infections. In young piglets, this additive was tested for its effect on growth performance, cecal microbial profiles, jejunal morphology, and gene expression. The diet did not affect performance traits, but increased jejunal villus height and altered the microbial structure in cecal digesta. The expression microarray revealed changes in the transcript level of 1378 genes studied in jejunal tissue. The altered genes were clustered in cellular homeostasis and development, protein synthesis, and immune-modulation pathways, suggesting that a mannan-rich diet can bring benefits in term of interstitial homeostasis (Fouhse et al. 2019).

## Nutrigenomics in poultry

### Supplementation with plant origin extracts

Poultry, most of which consists of chickens, is the farm animal with the largest number of breeding herds and individuals in the world, while chicken meat is also the most frequently eaten meat (https://ourworldindata.org/meat-and-seafood-production-consumption). Researchers and breeders are thus interested in optimizing the production of healthy animal origin products. Different approaches to dietary treatment of hens and chickens focus on proper growth and health status of the birds. The addition of phytonutrients to chicken feed is considered a natural means of protecting against infections. One study employed supplementation with carvacrol, cinnamaldehyde, and *Capsicum* oleoresin from day 7 after hatching, studying the transcriptome of intestinal intraepithelial lymphocytes with the microarray approach (Kim et al. 2010). Significant gene expression changes were observed in the group receiving the *Capsicum* oleoresin supplementation, where the genes involved in lipid metabolism, small molecule biochemistry, and cancer showed altered expression. In a later study of the same group, the same experimental design was applied with the addition of an oral challenge with *Eimeria acervulina*, a pathogen responsible for coccidiosis in chicken. While the supplementation resulted in improved body weight, the cinnamaldehyde-rich diet altered the expression of genes responsible for antigen presentation, humoral immune response, and inflammatory disease. This led to the assumption that natural phytonutrients can positively affect the host’s immune system and gut metabolic condition, and could be considered as an alternative to antibiotics in poultry feeding (Lillehoj et al. 2011). The search for natural components which could protect against coccidiosis, a very common disease induced by bacteria of the *Eimeria* genus, has also been taken up by other research groups. In relation to transcriptional changes in gut lymphocytes, *Curcuma longa*, anethole, and garlic metabolites have been tested as nutritional supplements. Three studies (Kim et al. 2013a, b, c) showed that multiple genes involved in inflammatory response were altered, indicating a potential immunoprotective role of plant extracts against avian coccidiosis. Therefore, it is highly recommended to look for natural nutrients that improve the health of birds that could eliminate antibiotics from poultry feeding resulting in healthier meat. The use of post-production plant waste is very popular in animal feeding and can bring many benefits both in breeding and environmental protection through the reuse of production waste. Olive mill wastewater—a waste product obtained during olive oil extraction—is rich in polyphenols and has already been used in chicken feeding. Its effect on the transcriptome profile of jejunum has also been investigated by Sabino et al. (2018b), who found that, in epithelial cells of the jejunum, 280 genes were differentially expressed in birds fed the experimental diet. The downregulated genes were mostly associated with the PPAR signaling pathway, with steroid biosynthesis, and with lipid metabolisms; the upregulated genes were involved in the regulation of viral processes (such as viral replication and viral regulation of the life cycle). It was concluded that the use of such waste material in animal feeding can bring benefits for small intestine functioning, consequently improving chicken health. In a later work by the same research group, a diet supplemented with oregano (which has antimicrobial, antifungal, and antioxidant properties) was given to chickens, and its effect on the hepatic transcriptome profile was investigated (Sabino et al. 2018c). A total of 129 genes showed differing expression profiles between the supplemented and control groups, with a prevalence of reduced transcription in animals fed the oregano addition. These downregulated genes were mostly involved in insulin-signaling pathways and fatty acid metabolism, suggesting a potential role of oregano supplementation in the reduction of fat deposition in broiler chickens.

### Supplementation with microelements

Rebel et al. (2006) studied the effect of supplementing hens’ diet with vitamins and minerals on the intestinal transcriptional profile of the offspring. They found that supplementation increased the mRNA level of genes associated with turnover and proliferation of intestinal cells, which was in agreement with the number of proliferating cells in the villus and transitional area in a supplemented group. The expression of genes that could affect metabolism, feed absorption, and intestinal immune system was also altered (Rebel et al. 2006). The effect of trivalent chromium supplementation in broiler chickens was studied by Pan et al. (2013) in terms of microRNA expression profile in muscle tissue, as well as its effect on protein synthesis. Chromium is an essential element in diet that is necessary for the correct metabolism of nucleic acid, lipids, and carbohydrates. It was found that chromium supplementation altered the total serum proteins and serum triglycerides, consequently affecting insulin action. Of the 57 microRNAs with altered expression in muscle tissue, 16 with enhanced transcription were involved in the growth and development of skeletal muscle (Pan et al. 2013). A study of the effect of methionine deficiency and its later supplementation in the diet of broilers was recently performed (Aggrey et al. 2018). The transcriptional profile of one-carbon metabolism genes was determined in the liver, duodenum, and two muscle tissue depots. The decreased level of methionine in the diet had a negative effect on growth and decreased the hepatic transcript of the methyltransferase (*MAT1A*) gene, which could potentially led to a reduction in SAM levels and an increase in oxidative stress. The low level of SAM in the duodenum and liver could possibly downregulate transcription of the glycine N-methyltransferase (*GNMT*) gene, which is essential for the conversion of SAM to SAH, and which consequently reflects cell methylation potential. Cysteine deficiency in chicken diet has also been studied. This amino acid is synthetized from methionine and is important for bird plumage quality. The deficient diet was employed to determine the effect on skin thickness, length of feather follicle, and hepatic transcript level of selected genes. Unbalanced cysteine levels in the diet resulted in reduced epidermis thickness and shorter feather follicles than in the case of the controls. The levels of expression of cystathionine beta synthase (*CBS*) and cystathionine-lyase (*CTL*) also increased, altering the transsulfuration pathway, which could lead to effect on thermoregulation (Vilar Da Silva et al. 2019). This study shows that not only main economical traits should be studied, but also features concerning animal welfare are important for effective production. The role of prebiotics in animal diets was studied by Sevane et al. (2014). Such dietary additives can have a wide range of benefits for health, enhancing immune functions, preventing infections, decreasing disease risk, and improving production traits, such as growth rate. Thus, dietary supplementation with inulin (isolated from chicory) in broiler chickens was used to determine the effect on the hepatic transcriptome profile. The authors of that study found that, of 148 differentially expressed genes, 104 were upregulated, while decreased expression was observed for 44 in the experimental group. The KEGG database indicates that the genes altered by inulin supplementation relate to metabolic pathways, and could thus alter the birds’ growth and performance, affect immune status, and be associated with lipid metabolism (Sevane et al. 2014).

## Summary

Dietary manipulation in farm animals focuses on the effect on production traits, such as growth, quality of animal products, and the animal’s health. Correct diet brings economic benefits, and for this reason nutrients that can improve animal breeding are sought. The main goal in animal breeding to produce healthy meat should be achieved through proper, balanced feeding, while the tested alternative nutrients should be carefully examined in terms of short- and long-term effects. Studies of gene expression analysis in response to dietary regimens are carried out because changes in biochemical parameters depend on particular gene transcripts. Thus, such experiments in beef cattle generally concentrate on obtaining the ideal fatty acid profile in muscle tissue, while in dairy breeds the milk yield and composition are the most important features that can be modulated by specific nutrition. Meat quality is essential in pig production, so many natural, plant-origin feed additives are available. As in pigs, animal health is very important in poultry, so supplements such as vitamins, amino acids, and prebiotics are used to affect transcription of genes that play a role in correct immune system function. Recently, the search for unconventional feed additives is very common, thus further studies with the use of e.g. post-production plant wastes in animal nutrition are needed. It must be keep in mind that the environmental factors, including nutrition, can induce completely different effects depending on such parameters as dosage, duration of diet regimen, physiological status of the animals, etc. what in consequence can led to discrepancy of the obtained results from similar experiments. Although transcriptomic studies have been conducted in livestock, very little is known concerning proteome level; thus, complex studies including transcript as well as protein levels should be undertaken. Also, the effect of nutrition on epigenetic mechanisms is still poorly understood in farm animals. DNA methylation, histone modifications, and noncoding RNA interactions are the main mechanisms that regulate genes expression, and future research into the effects of diets on these mechanisms is highly recommended. Cutting-edge technology, such as whole-genome bisulfite sequencing (WGBS), chromatin immunoprecipitation sequencing (ChIP-Seq), and global RNA-sequencing, is currently widely used for human and rodent nutrigenomic studies, and certainly will be used more frequently in other species. A comprehensive approach including genome functioning, physical parameters, and breeding goals should be undertaken during nutrigenomic studies.
